# Beliefs and Experiences of People in Australia Living With Chronic Pelvic Pain, and Associations Between Their Illness Perceptions and Pain

**DOI:** 10.1111/ajo.70182

**Published:** 2026-09-18

**Authors:** Emma Wise, Amelia Cornish, Michelle Franklin, Maria del Pilar Martin Garcia, Zoe Mills, Darren Beales

**Affiliations:** ^1^ School of Allied Health Curtin University Perth Australia

**Keywords:** health beliefs, healthcare providers, pelvic pain, sexual trauma, women's health

## Abstract

**Background:**

There are gaps in understanding females' beliefs related to chronic pelvic pain. Beliefs might be linked to illness perceptions and behaviours.

**Aims:**

(1) Explore beliefs and experiences of Australian females living with chronic pelvic pain, against categories based on clinical guidelines. (2) Explore associations between illness perceptions and pain experience (severity and interference).

**Materials and Methods:**

A community‐based, online cross‐sectional study targeting women with chronic pelvic pain in Australia (*n* = 465) was performed. Data collected included participant beliefs and experiences related to diagnosis, healthcare practitioner interactions, assessments undertaken and management. Associations were assessed between illness perceptions and separately for pain severity and pain interference.

**Results:**

64.3% experienced pain before 18 years of age, but only 36.4% first sought help before 18. Of those seen by a healthcare practitioner, 42.9% felt their pain was not validated and 45.6% were not asked about their personal beliefs regarding the cause of their pain. Of those consenting to answer sexual abuse questions, only 25.6% reported having been screened by healthcare practitioners for a history of sexual abuse. Controlling for confounders, more threatening illness perceptions were associated with higher pain severity (coefficient 0.07 (95% confidence interval 0.05 to 0.09), *p* < 0.001) and higher pain interference (coefficient 0.15 (95% confidence interval 0.12 to 0.18), *p* < 0.001).

**Conclusion:**

Participants with chronic pelvic pain reported delayed care seeking during adolescence, a lack of validation by healthcare practitioners when seeking care and suboptimal screening of sexual abuse. These issues contradict guideline‐based recommendations, might negatively impact illness perceptions, which could contribute to increased burden.

## 1 Introduction

Chronic pelvic pain (CPP) is pain lasting > 6 months in the lower abdomen/pelvis (including genitalia) [1]. It is multifactorial, potentially involving urological, gynaecological, gastrointestinal, neurological and/or musculoskeletal contributions [1]. Population prevalence estimates suggest 6%–27% of females worldwide are affected [2], a likely underestimation [3]. CPP can be associated with bladder/bowel, pelvic floor, sexual and gynaecological dysfunction and with adverse cognitive, behavioural and emotional outcomes [4]. Burden is substantial, affecting relationships, education and work, with significant healthcare and productivity costs [3].

Many women with CPP report challenges navigating healthcare systems. Obtaining a satisfactory diagnosis can be difficult [5, 6]. Communication difficulty is frequently encountered, including not feeling listened to, not being taken seriously and inadequate provision of information [6, 7, 8]. These difficulties lead to some women disengaging from care [9]. Less seems known about the beliefs of women with CPP, particularly illness perceptions. More threatening illness perceptions have been associated with greater disease severity in people with interstitial cystitis/painful bladder syndrome [10] and endometritis specifically [11]. Poorer illness perceptions may create further challenges for people seeking care [11] and might impede healthcare practitioners' (HCPs) ability to care for those living with CPP.

Clinical practice guidelines (CPGs) support management of complex disorders like CPP [12]. However, many do not reflect contemporary biopsychosocial pain frameworks [13]. Biopsychosocial care requires an understanding of the beliefs and experiences of those experiencing CPP. Increasing HCPs' understanding of patient beliefs and experiences can enhance patient‐therapist therapeutic relationships, improve adherence to treatment and optimise management outcomes [14].

We profiled women with CPP regarding beliefs and care experiences using CPG domains as a framework, aiming to identify gaps between recommendations and lived experience. We further aimed to assess associations between illness perceptions and pain severity and interference, adjusting for confounders.

## 2 Materials and Methods

### 2.1 Study Design

A cross‐sectional study was performed. Consumers contributed to questionnaire development and interpretation of findings. Curtin University Human Research Ethics Office provided ethical approval (HRE2021‐0329).

### 2.2 Recruitment and Participants

We recruited via Australian social media groups and private clinics. Inclusion criteria were self‐reported pelvic pain for > 6 months, age ≥ 18 years, assigned female at birth and residence in Australia. Exclusion criteria were pregnancy within 12 months, breast/pelvic cancer diagnosis or treatment within 12 months or sexually transmitted infection within 6 months.

### 2.3 Procedure

An online questionnaire was hosted on Qualtrics (XM, North Sydney, Australia). Potential participants were provided information on the study, and consented when progressing to the questionnaire itself. The questionnaire was informed by CPGs, particularly the European Association of Urology (EAU) guidelines on CPP [12]. This guideline is commonly referred to in Australian institutions and includes recommendations for a multidisciplinary and biopsychosocial approach, aligning with contemporary biopsychosocial management of pain disorders. The full questionnaire is provided as Data S1.

### 2.4 Consumer Engagement

Twelve females with CPP and 21 HCPs were engaged to review the draft questionnaire. Based on their feedback, questions were removed or modified to improve questionnaire continuity.

### 2.5 Variables

We collected demographics (including age, symptom onset, education/employment, residence, relationship status, hormonal status and sexual abuse history) and pain characteristics (related to menstrual timing and dyspareunia). Pain was measured with the Brief Pain Inventory (BPI) (severity and interference) (used under licence: www4.mdanderson.org/symptomresearch). Illness perceptions were assessed with the Brief Illness Perception Questionnaire (BIPQ), where a higher score represents a more threatening view of one's illness.

Participants were asked if they had seen HCPs for CPP, their age when they first did so, what type of HCPs they had seen (and if this was helpful), what type of assessments were performed, if they believed they required further investigation, if they had been screened about a history of sexual abuse and if they felt a history of sexual abuse was related to their CPP. Participants were provided a list of potential mechanisms underlying CPP and asked which mechanisms might cause CPP and if they believed those selected mechanisms cause their CPP. Participants were asked about barriers to receiving care.

Aligned to CPGs, participants were asked about their experiences related to assessment/diagnosis, communication/support and treatment. For example they were asked if they had their pain legitimised, if they were asked their personal beliefs of the cause of their symptoms, if they received a diagnosis and if they felt they needed further investigations. Responses were on a 5‐point scale: strongly agree, somewhat agree, neither agree nor disagree, somewhat disagree or strongly disagree. Participants were given a list of potential treatment strategies and asked if that treatment had been used and was helpful.

### 2.6 Data Analysis

We excluded respondents who completed < 80% of the questionnaire (not including the sexual abuse questions which were optional). Analyses were performed with STATA/BE 17.0 (StataCorp, College Station, TX, USA).

Descriptive statistics were reported for all variables. Participant agreement with contemporary guideline informed recommendations for the management of CPP were presented in a heatmap. Univariable quantile regression was used to assess the association between participant characteristics, their beliefs and their experiences separately for the BPI pain severity and interference scores. Because age was associated with the BPI scores, all other univariable associations were adjusted for age. Then, quantile regression was used to test the association between the BPIQ with the BPI severity and interference scores, adjusting for potential derived from factors with significant univariable associations.

## 3 Results

### 3.1 Participant Characteristics

**FIGURE A1:**
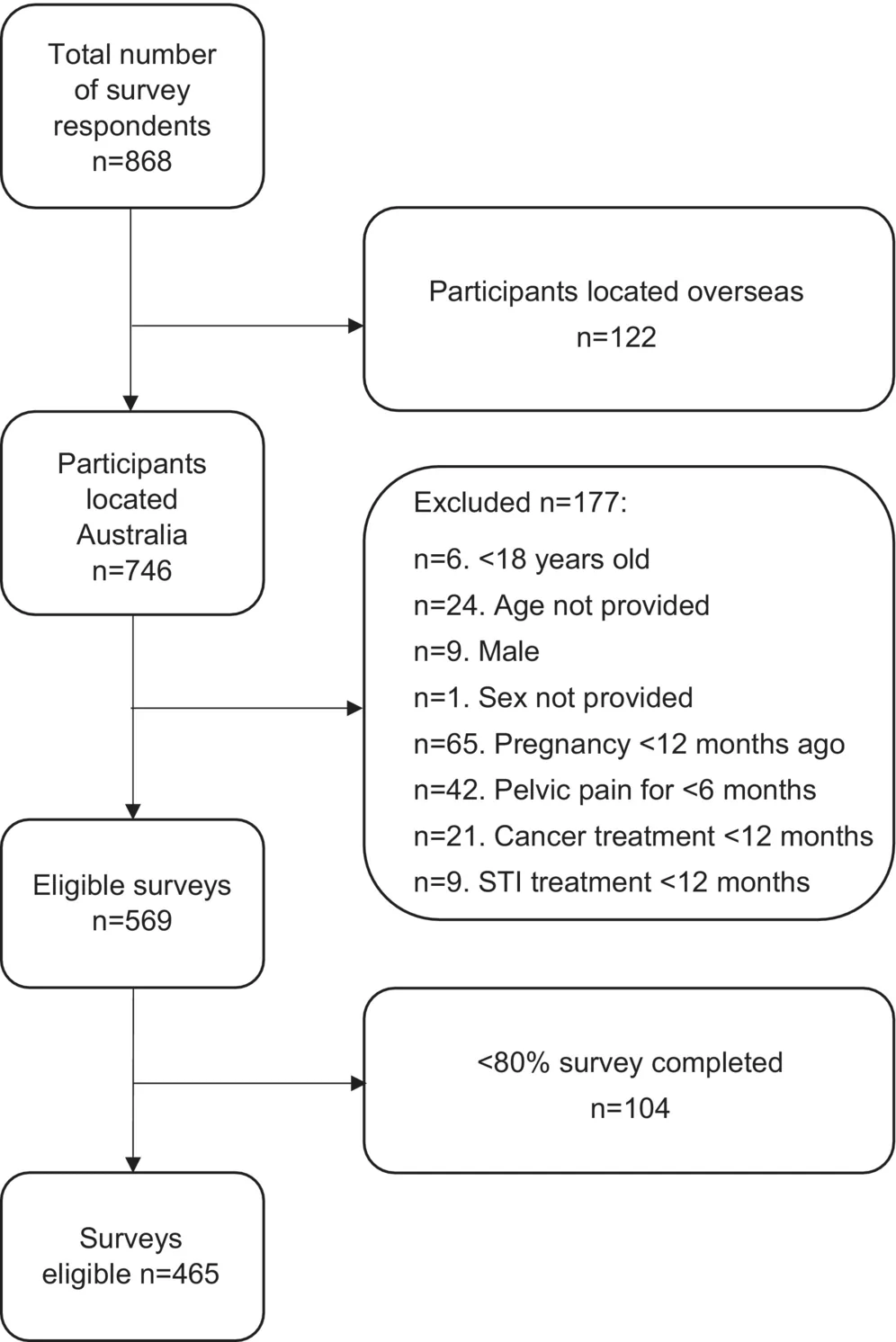
Flow chart for participant inclusion.

Four hundred and sixty‐five Australian females were included (Figure A1). Participant characteristics are summarised in Table 1. The majority were Caucasian (92.3%), median age of 29 (range 18–75) years, with 64.3% reporting pain onset before age 18. Most (85.6%) experienced pain both between and during menstruation. A third reported a history of sexual abuse.

**TABLE 1:** Participant characteristics and overview of their interaction with healthcare (*n* = 465).

Characteristic	Median (IQR) or *n* (%)	Experiences and beliefs	*n* (%)
*Age*	29 (24–35)	*Seen Health Care Practitioner, yes*	445 (95.7)
*Age at onset of pain*		*Age first saw Health Care Practitioner* ^e^	
Under 12	42 (9.0)	Under 18	162 (36.4)
12–17	257 (55.3)	18–25	172 (38.6)
18–25	96 (20.6)	26–35	83 (18.6)
26 and older	70 (15.1)	36 and Older	28 (6.3)
*Ethnicity*		*Health Care Practitioner seen*	
Caucasian	429 (92.3)	General Practitioner	442 (95.1)
Non‐Caucasian	36 (7.7)	Gynaecologist	408 (87.7)
*Religious*		Urologist	47 (10.1)
Religious	81 (17.4)	Colorectal Surgeon	33 (7.1)
Non‐religious	384 (82.6)	Gastroenterologist	150 (32.3)
*Highest level of education*		Pain Specialist	121 (26.0)
High school	55 (11.8)	Psychologist	179 (38.5)
Technical education	100 (21.5)	Physiotherapist	268 (57.6)
University or higher	310 (66.7)	Dietitian	103 (22.2)
*Employment (* *n* *(%))*		Sexologist	24 (5.2)
Full time paid	210 (45.2)	Alternative Medicine Practitioner	196 (42.2)
Part time paid	181 (38.9)	None listed	9 (1.9)
Unemployed	28 (6.0)	*Received a physical examination* ^e^ *, yes*	422 (90.8)
Disability pension	10 (2.2)	*Investigations performed*	
Other	36 (7.7)	Blood tests	331 (71.2)
*Location*		Urinalysis	273 (58.7)
Metropolitan	337 (72.5)	Vaginal swabs	258 (55.5)
Regional	122 (26.2)	Biopsy	183 (39.4)
Remote	6 (1.3)	Imaging	379 (81.5)
*Relationship status*		Surgery	295 (63.4)
Single	123 (26.5)	Scopes	200 (43.0)
Married/Defacto	316 (68.0)	Other	47 (10.1)
Divorced	10 (2.2)	None	5 (1.1)
Other	16 (3.4)	*Believe they require further investigations or procedures* ^f^	
*Sexual abuse* ^a^, ^b^		No	120 (25.8)
Sexually abused	145 (33.7)	Yes	259 (55.7)
Not sexually abused	267 (62.1)	Unsure	83 (17.9)
Prefer not to answer	16 (3.7)	*Asked if have experienced sexual abuse* ^a^, ^b^, yes	110 (25.6)
*Hormonal status*		*Barriers to receiving care*	
Breastfeeding	6 (1.3)	Long waitlists	241 (51.8)
Menopausal or post‐menopausal	23 (5.0)	Lack of information to make a decision about care	224 (48.2)
Hormonal replacement therapy^c^	25 (5.4)	Time poor and unable to commit to management options	74 (15.9)
Hormonal therapy^d^	260 (55.9)	Financial restraints and cost of management	270 (58.1)
Normal menses	94 (20.2)	Lack of access to multiple Health Care Practitioners	164 (35.3)
Irregular menses	94 (20.2)	Not understanding who I should see	190 (40.9)
Amenorrhea	22 (4.7)	Not understanding my condition	208 (44.7)
Nil hormonal treatmentor unsure	88 (18.9)	Not willing to try again as management has not been helpful previously	119 (25.6)
*Pain and Menstruation*		No barrier	25 (5.4)
Pain with menstruation only	18 (3.9)		
Pain between menstruation only	17 (3.7)		
Pain with and between menstruation	398 (85.6)		
*Pain with sexual intercourse, yes*	364 (78.3)		
*Brief Pain Inventory—severity*	3.8 (2.2–5.2)	*Brief Illness Perception Questionnaire*	51 (44–59)
*Brief Pain Inventory—interference*	4.9 (2.6–7.0)		

Abbreviation: IQR, inter quartile range.

^a^ Did not consent to answering question *n* = 35.

^b^ Missing *n* = 2.

^c^ Used for menopause or as feminising/masculinising.

^d^ Oral contraceptive pill, Implanon, Mirena, Zoladex.

^e^ Missing *n* = 20.

^f^ Missing *n* = 3.

#### 3.1.1 Participant Healthcare Experiences

Table 1 provides a broad overview of participants' interaction with healthcare. Half (55.7%) believed they needed further investigations/procedures, despite already undergoing many assessment procedures. Barriers to care related to limitations in access and limited understanding of their condition and management options. Most participants had seen a general practitioner (95%) and/or gynaecologist (88%) for their CPP, with 58% consulting with a physiotherapist. Global rating of helpfulness of seeing these HCPs varied from 46% to 68% (Table A1). Of the 428 that answered questions surrounding sexual abuse, 25.6% reported they were screened for this by HCPs.

#### 3.1.2 Participant Beliefs on CPP Causes

Participants' beliefs regarding potential causes of CPP were expansive (Table 2). Pathology (77%) and menstruation (61%) were the most frequently cited causes by participants to explain their own pain.

**TABLE 2:** Participants beliefs regarding causes of chronic pelvic pain (*n* = 465).

Causes of chronic pelvic pain	Believe could cause chronic pelvic pain	Believe causes their chronic pelvic pain
	*n* (%)	*n* (%)
Bladder	218 (46.9)	107 (23.0)
Bowel	235 (50.5)	168 (36.2)
Uterus	244 (52.5)	236 (50.8)
Ovaries	242 (52.0)	236 (50.8)
Pelvic floor muscles	241 (51.8)	257 (55.3)
Ligaments/Bones	180 (38.7)	68 (14.6)
Stress	196 (42.2)	190 (40.9)
Anxiety/Depression	159 (34.2)	125 (26.9)
Fatigue	146 (31.4)	86 (18.5)
Nerves	207 (44.5)	165 (35.5)
Infection	201 (43.2)	12 (2.6)
Immune system	171 (36.8)	54 (11.6)
Menstruation	253 (54.4)	283 (60.9)
Cancer	200 (43.0)	5 (1.1)
Pathology	268 (57.6)	359 (77.2)
Syndrome (IBS, BPS)	233 (50.1)	132 (28.4)
Inflammation	227 (48.8)	245 (52.7)

*Note:* Pathology example: Endometriosis, Crohn's Disease, Interstitial Cystitis.

Abbreviations: BPS, bladder pain syndrome; IBS, irritable bowel syndrome.

#### 3.1.3 Participant Experience of Contemporary Guideline Care

**FIGURE 1:**
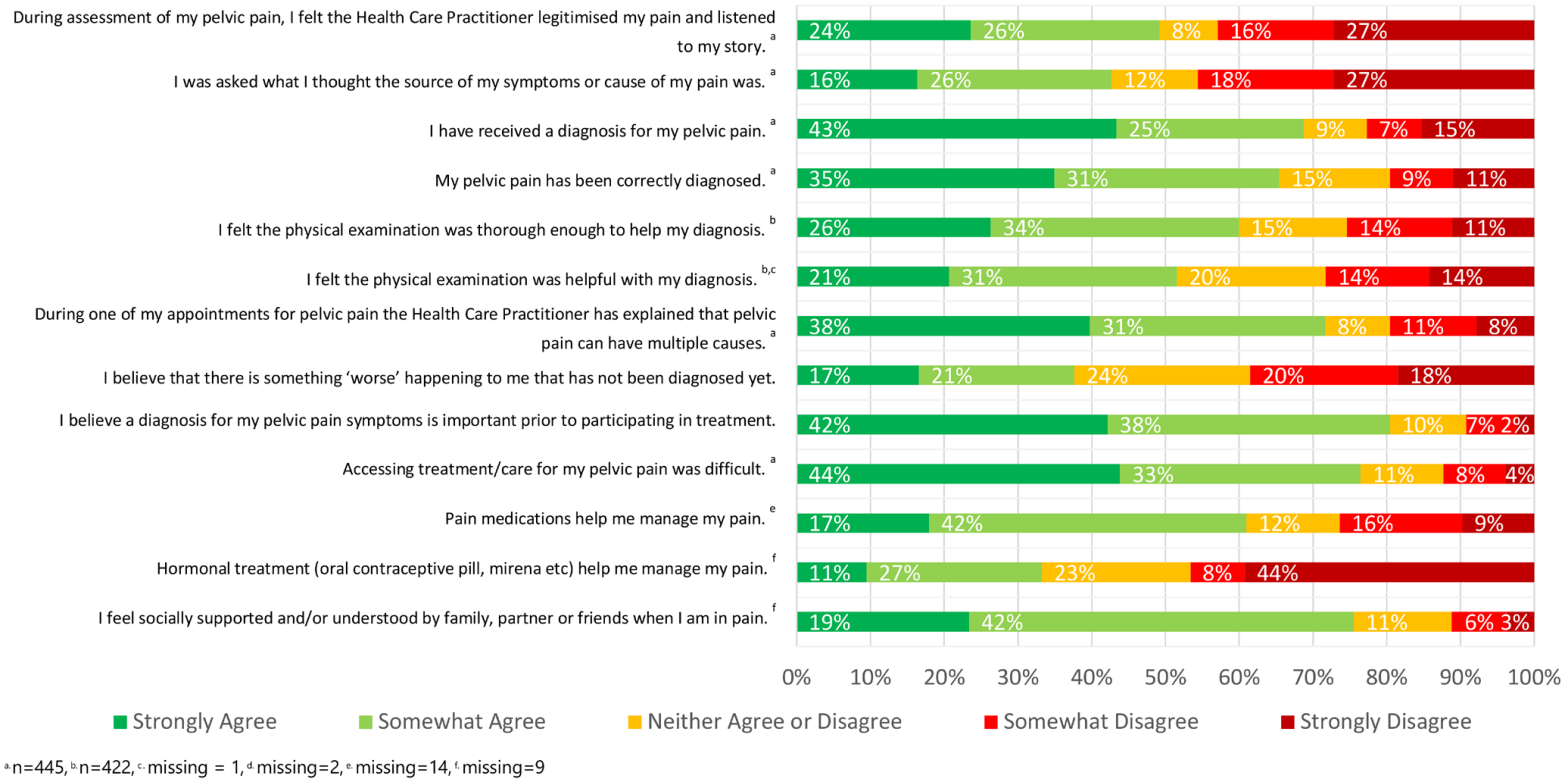
Participants' opinion of their own circumstances in regard to contemporary guideline recommendations for management of chronic pelvic pain (*n* = 465).

Figure 1 provides indication of participants’ opinion of their own circumstances regarding CPGs recommendations. Many participants did not feel listened to or legitimised (43%) and were not asked about their own opinion of the cause of their symptoms (46%). 66% felt their diagnosis was correct, though 38% believed that something ‘worse’ was happening. 59% found pain medication helpful, though this was reduced to 38% for hormone therapy. 61% felt socially supported. Access to care was difficult for 77% of participants.

#### 3.1.4 Participant Experience of CPP Management

Additional information on whether management had been helpful for the participants is summarised in Table A2. Some of the most highly rated helpful management strategies were heat/cool packs (85%), low intensity exercise‐based management (42% to 49%), breathing/relaxation exercises (49%), physiotherapy prescribed exercises (43%), activity avoidance (43%) and surgery (41%).

### 3.2 Association of the BIPQ With the BPI‐Severity

In univariable modelling, a history of sexual abuse, the timing of pain with regard to menstruation, pain during sex, a belief that additional investigations were required, along with age, were associated with pain severity (Table 3). Adjusting for these confounders, a higher score on the BIPQ indicating more threatening illness perceptions was associated with higher BPI severity (*n* = 351, coefficient 0.07 (95% CI 0.05–0.09), *p* < 0.001).

**TABLE 3:** Univariable associations between participant characteristics, beliefs and experiences with the Brief Pain Inventory (BPI) pain severity and pain interference scores (*n* = 465).

	Univariable associations	
	BPI severity	BPI interference
Age	−0.03 (−0.06 to −0.00), 0.049	−0.06 (−0.11 to −0.02), 0.008
Age at onset of pain		
Under 12	ref	ref
12–17	−0.68 (−1.58 to 0.23), 0.142	−1.07 (−2.51 to 0.37), 0.145
18–25	−1.00 (−2.01 to 0.01), 0.052	−2.35 (−3.96 to −0.75), 0.004
26 and older	−0.39 (−1.51 to 0.73), 0.491	−0.45 (−2.23 to 1.34), 0.623
Ethnicity		
Caucasian	ref	ref
Non‐Caucasian	0.58 (−0.35 to 1.52), 0.221	−0.04 (−1.59 to 1.51), 0.964
Religious		
Religious	ref	ref
Non‐religious	−0.20 (−0.86 to 0.46), 0.553	0.36 (−0.73 to 1.45), 0.514
Highest level of education		
High school	ref	ref
Technical education	−0.01 (−0.97 to 0.95), 0.981	−0.14 (−1.68 to 1.40), 0.855
University or higher	−0.74 (−1.57 to 0.10), 0.083	−1.30 (−2.64 to 0.04), 0.057
Location XX		
Metropolitan	ref	ref
Rural/Remote	0.15 (−0.46 to 0.76), 0.626	0.08 (−0.83 to 1.00), 0.859
Sexual Abuse^a^, ^f^		
Sexually abused	ref	ref
Not sexually abused	−0.89 (−1.44 to −0.33), 0.002	−1.78 (−2.65 to −0.91), 0.000
Pain and Menstruation^b^		
Pain with menstruation only	ref	ref
Pain between menstruation only	−0.22 (−1.80 to 1.35), 0.780	−1.11 (−3.88 to 1.66), 0.430
Pain with and between menstruation	−1.87 (−3.05 to −0.69), 0.002	−2.99 (−5.07 to −0.91), 0.005
Pain with sexual intercourse^c^		
No	ref	ref
Yes	1.27 (0.67 to 1.86), 0.000	1.72 (0.55 to 2.88), 0.004
Brief Illness Perception Questionnaire	0.09 (0.07 to 0.11), 0.000	0.15 (0.13 to 0.18), 0.000
Age first saw Health Care Practitioner^d^		
Under 18	ref	ref
18–25	−0.5 (−1.05 to 0.05), 0.077	−0.83 (−1.85 to 0.18), 0.105
26–35	0.16 (−0.57 to 0.82), 0.725	0.59 (−0.68 to 1.86), 0.358
36 and Older	−0.43 (−1.67 to 0.82), 0.503	0.71 (−1.56 to 2.98), 0.537
Believe they require further investigations or procedures^e^		
No	ref	ref
Yes	1.53 (0.98 to 2.08), 0.000	2.25 (1.26 to 3.25), 0.000
Unsure	1.44 (0.73 to 2.14), 0.000	1.37 (0.09 to 2.64), 0.036

*Note:* Univariable association via quantile regression (coefficient (95% confidence interval), *p*); associations adjusted for Age.

^a^ *n* = 412.

^b^ *n* = 426.

^c^ *n* = 432.

^d^ *n* = 445.

^e^ *n* = 462.

^f^ Responses of prefer not to answer not included in analysis.

### 3.3 Association of the BIPQ With the BPI‐Interference

In univariable modelling a history of sexual abuse, age of symptom onset, the timing of pain with regard to menstruation, pain during sex, a belief that additional investigations were required along with age were associated with pain interference (Table 3). Adjusting for these confounders, a higher score on the BIPQ indicating more threatening illness perceptions was associated with a higher BPI interference (*n* = 351, coefficient 0.15 (95% CI 0.12–0.18), *p* < 0.001).

### 3.4 Consumer Feedback

Three women with CPP were engaged in feedback of the results. All agreed that the study was worthwhile and increased the voice of consumers. They indicated the results reflected aspects of their own experiences. They commented on the potential for education in school students, including males, that included the impacts related to sexual abuse. Questions were raised about how this information might assist meaningful change for consumers.

## 4 Discussion

### 4.1 Principle Findings

This study summarised beliefs and care experiences of women with CPP. While many experiences aligned with guideline recommendations for referral, investigations and medical treatment, key gaps remained. Although 64.3% reported pain onset before age 18, only 36.4% sought care before 18. More than 2/5 felt their pain was not validated and were not asked about their beliefs regarding cause. Despite 1/3 disclosing sexual abuse, only 25.6% reported being screened. More than half believed further investigations were needed, and 77% attributed symptoms to pathology. More threatening illness perceptions were associated with higher pain severity and interference.

### 4.2 Comparisons to the Literature

Guidelines recommend adolescents should be encouraged to seek support before symptoms become severe [12, 15]. Adolescents can use suboptimal self‐management strategies if not seen [16]. If care is delayed it can lead to a negative impact of CPP on study and extracurricular activities [16]. Education programs may reduce the culture of secrecy that is often associated with CPP, and provide information on what is normal versus when to seek help [14, 17]. Online delivery of such programs may provide trustworthy menstrual health literacy information for adolescents [18].

Guidelines suggest that healthcare practitioners can treat CPP with a working diagnosis, not requiring repeat or unnecessary investigations [15, 19]. Our participants may not be aware of this, as many thought they required more investigations (55.7%) which might include surgery. Surgery can assist, though it has risks and a compounding effect on CPP if the pain is not adequately managed post‐surgery [15, 19].

The EAU guidelines recommend that HCPs provide validation of the patient's pain [12]. Pain validation and assessing beliefs regarding the cause of pain are critical to developing a strong therapeutic alliance, underpinning meaningful and effective management for chronic pain. Our results show some disconnect between CPGs and patient experiences which are reflected elsewhere. When adolescents seeking care are told their menstrual pain is normal and dismissed, it can cause more concerns as an adult [18]. When HCPs fail to convey understanding of the patient's symptoms and the impact on their quality of life, patient dissatisfaction can occur [14, 20, 21]. Allowing adequate time to explore and address these issues at the initial consultation can reduce follow up needs, saving time and money [20, 21]. Participants' lack of a sense of validation could reflect lower confidence of some HCPs in managing these disorders and not always assessing patient beliefs and goals [22]. Evaluation of patient‐therapist interactions could shed more light on this and help optimise validation strategies.

Disclosure of CPP can be difficult. Failure to validate a patient's pain experience inhibits patients from discussing their concerns as to the cause of their pain [14, 23]. This may lead to missed opportunities for education particularly in relation to a diagnosis, missing the opportunity to allay potential fears and catastrophising beliefs [20, 24]. Comprehensive chronic pain education is at the forefront of management, including messaging that pain can be experienced in the absence of pathology or tissue damage. Our study showed 77% felt their pain was due to pathology; this could indicate a gap in chronic pain knowledge.

The prevalence of sexual abuse history in our participants was 1/3, which is higher than the general prevalence of approximately 15% [25]. Guidelines suggest screening all those with CPP for a history of sexual abuse [12]. This has not been the case with our participants. In univariable associations, a history of sexual abuse was associated with greater levels of pain severity and interference (Table 3). Somatisation and dissociation have been described as part of the trauma response, which could play a role in altered nociceptive processing related to CPP [14, 23]. Obstetrician‐Gynaecologists understand screening for sexual abuse is important; however, they regularly avoid this, citing inadequate training [26]. Missed screening is a missed opportunity for adequate care. Training in trauma‐informed care is salient for HCP enabling communication, avoiding discrediting the patient's experiences and assisting informed decision making for assessment and treatment options [24, 27]. If trauma is not acknowledged nor identified, invasive investigations and treatment methods may trigger traumatic memories and induce further trauma [24]. All educators need to be familiar with the World Health Organisation's Comprehensive Sexuality Education (www.who.int/news‐room/questions‐and‐answers/item/comprehensive‐sexuality‐education) to help conceptualise positive sexuality from a young age and to educate on when to seek help.

More threatening illness perceptions were associated with increased pain severity and interference. This is consistent with prior findings in people with interstitial cystitis/painful bladder syndrome [10] and endometriosis specifically [11]. The beliefs and experience documented here reflect elements of illness perception. Further work is required to understand if addressing beliefs might improve illness perceptions. This may align to suggestions for managing illness perceptions in disorders such as breast cancer‐related lymphedema risk management [28, 29].

### 4.3 Clinical Implications

The complexity of CPP complaints necessitates person‐centred, holistic care [1, 4]. Our results highlight areas for practitioner focus. Firstly, understanding the illness perceptions of patients may be valuable. Short of providing patients the BIPQ, HCPs may ask directly about a patient's personal understanding of the cause of their symptoms. Acknowledgement of this provides a focus for individual patient education. It may negate patients not feeling listened to and validated as per our cohort. Further, screening for sexual abuse using the principle of trauma‐informed care would negate a gap experienced by many patients. Future research focusing on these areas would aim to address these gaps and could improve patient experiences and ultimately reduce CPP burden. The consumer group engaged in reviewing the study results highlighted the need for people with CPP to have more understanding, so they are more confident in their patient‐clinician interactions.

### 4.4 Strengths, Limitations and Methodological Considerations

Strengths included a relatively large community‐dwelling sample, profiling across key CPG domains, controlling for multiple confounders and continuous consumer engagement throughout the study. However, online recruitment limited the sample to those with online access. People from remote regions were under‐represented. The sample was highly educated and may not fully reflect the experience of people with lower levels of education. Thus, the results may not be applicable to people who are more socioeconomically disadvantaged. The meaning of sexual abuse was not well defined and would benefit from further research. Our cross‐sectional data cannot determine cause and effect relationships.

In summary, this study shares the patient's perspective and highlighted gaps in practice related to seeking care as an adolescent, validation of the patient's experience and suboptimal screening of sexual abuse. These issues might influence illness perceptions, which were found to have a negative impact upon individual burden related to CPP in this study.

## Funding

Use of the Brief Pain Inventory was funded by Curtin University.

## Conflicts of Interest

The authors declare no conflicts of interest.

## Supporting information


**Data S1:** Full questionnaire.

## Data Availability

The data that support the findings of this study are available from the corresponding author upon reasonable request.

## Appendix A

**TABLE A1:** Demographic and chronic pelvic pain characteristics of Australian study participants with comparison to the excluded international cohort who completed greater than 80% of the questionnaire (reported as median (interquartile range) or *n* (%)), and the Australian participants who completed less than 80% of the questionnaire.

Characteristic	Final Cohort of Australian Participants (*n* = 465)	Excluded International Participants (*n* = 72)	*p*	Excluded Participants < 80% Questionnaire Completed (*n* = 104)	*p*
Age	29 (24–35)	29 (25–33)	0.831	28 (24–35)	0.987
Age at onset of pain					
Under 12	42 (9.0)	5 (6.9)	0.016	10 (11.2)	0.799
12–17	257 (55.3)	30 (41.7)		45 (50.6)	
18–25	96 (20.6)	27 (37.5)		21 (23.6)	
26 and older	70 (15.1)	10 (13.9)		13 (14.6)	
Ethnicity					
Caucasian	429 (92.3)	60 (83.3)	0.014	84 (88.4)	0.219
Non‐Caucasian	36 (7.7)	12 (16.7)		11 (11.6)	
Religious					
Religious	81 (17.4)	44 (61.1)	< 0.001	15 (15.8)	0.701
Non‐religious	384 (82.6)	28 (38.9)		80 (84.2)	
Highest level of education					
High school	55 (11.8)	9 (12.5)	0.327	13 (13.7)	0.686
Technical education	100 (21.5)	10 (13.9)		17 (17.9)	
University or higher	310 (66.7)	53 (73.6)		65 (68.4)	
Employment					
Full time paid	210 (45.2)	45 (62.5)	0.022	57 (60.0)	0.095
Part time paid	181 (38.9)	13 (18.1)		26 (27.4)	
Unemployed	28 (6.0)	8 (11.1)		4 (4.2)	
Disability pension	10 (2.2)	2 (2.8)		1 (1.0)	
Retired	5 (1.1)	1 (1.4)		3 (3.2)	
Volunteer	2 (0.4)	0 (0)		—	
Other	29 (6.2)	3 (4.2)		4 (4.2)	
Location					
Metropolitan	337 (72.5)	37 (51.4)	< 0.001	78 (82.1)	0.146
Regional	122 (26.2)	25 (34.7)		16 (16.8)	
Remote	6 (1.3)	10 (13.9)		1 (1.0)	
Relationship status					
Single	123 (26.5)	28 (38.9)	0.294	36 (37.9)	0.120
Married/Defacto	316 (68.0)	41 (56.9)		55 (57.9)	
Divorced	10 (2.2)	1 (1.4)		—	
Widow	1 (0.2)	0 (0)		—	
Other	15 (3.2)	2 (2.8)		4 (4.2)	
Sexual abuse^a^, ^b^					
Sexually abused	145 (33.7)	19 (27.9)	0.828	Not completed	
Not sexually abused	267 (62.1)	40 (58.8)			
Prefer not to answer	16 (3.7)	3 (4.4)			
Pain and Menstruation					
Pain with menstruation only	18 (3.9)	6 (8.3)	0.218	5 (5.7)	0.016
Pain between menstruation only	17 (3.7)	2 (2.8)		9 (10.2)	
Pain with and between menstruation	398 (85.6)	59 (81.9)		67 (76.1)	
Pain with sexual intercourse (yes)	364 (78.3)	55 (76.4)	0.734	54 (60.7)	0.001
Brief Pain Inventory—severity	3.75 (2.25–5.25)	3.75 (2.5–5.75)	0.627	2.75 (1.75–4.25)	0.002
Brief Pain Inventory—interference	4.86 (2.57–7.00)	5.79 (3.5–7.36)	0.091	4 (1.71–5.86)	0.166
Brief Illness Perception Questionnaire	51 (44–59)	57.5 (49–64.5)	< 0.001	50 (45–53)	0.459

*Note:* Comparisons Kruskal–Wallis test or Chi‐square test.

^a^ Did not consent to answering question: Australian's *n* = 35, Internationals *n* = 4.

^b^ Missing data: Australian's *n* = 2, Internationals *n* = 6.

**TABLE A2:** Treatment strategies that participants reported to be helpful for managing their chronic pelvic pain (*n* = 465).

Treatment strategies	Helpful *n* (%)
Avoid activity, exercise, tasks	199 (42.8)
Meditation/Mindfulness	143 (30.8)
Breathing or relaxation exercises	226 (48.6)
Psychological/behavioural therapy	89 (19.1)
Heat or cool pack	394 (84.7)
Low impact exercise	195 (41.9)
High intensity exercise	38 (8.2)
Strengthening exercise	99 (21.3)
Stretching/yoga	199 (42.8)
Playing sport	22 (4.7)
Dancing	25 (5.4)
Bladder/bowel habits	157 (33.8)
Dietary changes	163 (35.1)
Socialising or connection with family of friends	90 (19.4)
Pelvic physiotherapist prescribed exercises	198 (42.6)
Massage therapy	96 (20.7)
Electrical stimulation	109 (23.4)
Acupuncture/Chinese medicine	52 (11.2)
Naturopathy herbal medicine	51 (11.0)
Tai Chi/Qi Gong	4 (0.9)
Alcohol	34 (7.3)
Cannabis or hemp oil	72 (15.5)
Surgery or procedures	192 (41.3)
Hobbies	58 (12.5)
Treatment strategies count	
1–5	213 (45.8)
6–10	177 (38.1)
11–15	53 (11.4)
16–22	8 (1.7)
None of the strategies listed	14 (3.0)

*Note:* Low impact exercise examples: walking, cycling, swimming, light weights; High intensity exercise examples: running, HIIT, cardio, aerobics, gym; Strengthening exercise examples: pilates, weights, gym; Dietary changes examples: FODMAP, low sugar, low processed food, gluten free, vegan; Pelvic physiotherapist prescribed exercises examples: soft tissue massage of pelvic muscles, dilator therapy, electrical stimulation, soft tissue massage of pelvic muscles, dilator therapy, electrical stimulation, dry needling; Electrical stimulation examples: TENS or implanted device; Hobbies examples: music, art, drawing etc.
