# Supplementary material for: Beliefs and Experiences of People in Australia Living With Chronic Pelvic Pain, and Associations Between Their Illness Perceptions and Pain
Source: Aust N Z J Obstet Gynaecol. 2026 Sep 18;66(5):e70182. doi: 10.1111/ajo.70182 (PMC13589048; doi:10.1111/ajo.70182)
Supplement: Supplementary file 1 [file AJO-66-0-s001.docx]

**Supplementary Material:** Full questionnaire

Beliefs and experiences of people living with pelvic pain and their health care management.

Start of Block: Default Question Block

Start of Block: Block 1

We are interested in understanding what people know about pelvic pain and their experiences with management. There are guidelines for health care professionals to assist management of pelvic pain. We want to know if these guidelines align to patient expectations.    Chronic pelvic pain is pain between the belly button and upper thighs (as seen in below picture-circled in purple) and it feels like it comes from the pelvic organs, genitals, bones or other tissues such as the muscles or ligaments for 6 months or longer.   If you agree to participate, you will be asked to complete the following online questionnaire that involves answering a few questions about yourself and your experiences.  A full participant information sheet can be found by clicking on this link: Participant Information Sheet
 This questionnaire is anticipated to take approximately 20 minutes. You can cease the questionnaire at anytime or withdraw from the study at anytime. If you become uncomfortable or distressed you can stop, save your answers and complete at a later time.    The questionnaire is being conducted using Qualtrics, with the server being hosted in a secure data centre located in Australia. All information collected will be anonymous and confidential.   **By moving to the next page, you are consenting to participate in this study.**
  If you have any further questions please contact: m.martingarcia@postgrad.curtin.edu.au   Curtin University Human Research Ethics Committee (HREC) has approved this study (HREC number 2021-0329).  Should you wish to discuss the study with someone not directly involved, in particular, any matters concerning the conduct of the study or your rights as a participant, or you wish to make a confidential complaint, you may contact the Ethics Officer on (08) 9266 9223 or the Manager, Research Integrity on (08) 9266 7093 or email hrec@curtin.edu.au.

| Page Break |  |
| --- | --- |

What is your age?

- Under 18
- 18
- 19
- 20
- 21
- 22
- 23
- 24
- 25
- 26
- 27
- 28
- 29
- 30
- 31
- 32
- 33
- 34
- 35
- 36
- 37
- 38
- 39
- 40
- 41
- 42
- 43
- 44
- 45
- 46
- 47
- 48
- 49
- 50
- 51
- 52
- 53
- 54
- 55
- 56
- 57
- 58
- 59
- 60
- 61
- 62
- 63
- 64
- 65
- 66
- 67
- 68
- 69
- 70
- 71
- 72
- 73
- 74
- 75
- 76
- 77
- 78
- 79
- 80
- 81
- 82
- 83
- 84
- 85
- 86
- 86
- 88
- 89
- 90
- 91
- 99
- 93
- 94
- 95
- 96
- 97
- 98
- 99
- 100 and Older

What sex were you assigned at birth?

- Male
- Female

Have you had a baby or been pregnant in the last 12 months?

- Yes
- No

Have you had pelvic pain for more than 6 months?

- Yes
- No

Have you had a diagnosis/treatment of; breast or pelvic (bladder, bowel, uterus, cervix, genitals) cancer in the last 12 months?

- Yes
- No

Have you been diagnosed with a Sexually Transmitted Infection (STI) in the past 6 months?

- Yes
- No

| Page Break |  |
| --- | --- |

What is your ethnicity or cultural background?

- Caucasian
- Non-Caucasian

Do you practice any religion?

- Yes
- No

What is your highest level of education?

- High school
- TAFE or certificate
- University degree

What is your employment status?

- Paid full time employment
- Paid part time or casual employment
- Unemployed
- Disability Pension
- Retired
- Volunteer
- Other

Where do you live?

- Metropolitan
- Regional
- Remote

What is your relationship status?

- Single
- Married/Defacto
- Divorced/Separated
- Widow
- Other

What is your current hormonal status? You may select more than one

- Lactating (breastfeeding)
- Menopausal
- Post menopausal
- Using hormonal replacement therapy (for example; oestrogen gel/patches/tablets/creams)
- Using feminising or masculinising hormones
- Oral contraception pill or mini pill
- Mirena
- Implanon (small arm implant) or Depo Provera (injection)
- Zoladex (Goserelin)
- Normal regular period
- Absent period
- Irregular period
- Unsure
- Not using any hormonal treatment

| Page Break |  |
| --- | --- |

What age did you start to experience your pelvic pain?

- Less than 12 years old
- 12-17
- 18
- 19
- 20
- 21
- 22
- 23
- 24
- 25
- 26
- 27
- 28
- 29
- 30
- 31
- 32
- 33
- 34
- 35
- 36
- 37
- 38
- 39
- 40
- 41
- 42
- 43
- 44
- 45
- 46
- 47
- 48
- 49
- 50
- 51
- 52
- 53
- 54
- 55
- 56
- 57
- 58
- 59
- 60
- 61
- 62
- 63
- 64
- 65
- 66
- 67
- 68
- 69
- 70
- 71
- 72
- 73
- 74
- 75
- 76
- 77
- 78
- 79
- 80
- 81
- 82
- 83
- 84
- 85
- 86
- 86
- 88
- 89
- 90
- 91
- 99
- 93
- 94
- 95
- 96
- 97
- 98
- 99
- 100 and Older

Do you experience pelvic pain with menstruation (with your period)?

- Yes
- No
- Not applicable

Do you experience pelvic pain between menstruation (between period times of month)?

- Yes
- No
- Not applicable

Do you experience pelvic pain with sexual intercourse?

- Yes
- No
- Not applicable

| Page Break |  |
| --- | --- |

Brief Pain Inventory (Short Form)

Throughout our lives most of us have had pain from time to time (such as minor headaches, sprains and toothaches). Have you had pain other than these everyday kinds of pain today?

- Yes
- No

Item from Brief Pain Inventory © Copyright 1991 – Charles S. Cleeland, PhD Pain Research Group - All rights reserved.

| Page Break |  |
| --- | --- |

Please rate your pain by selecting the one number that best describes your pain at its WORST in the past 24 hours.

- 0 No pain
- 1
- 2
- 3
- 4
- 5
- 6
- 7
- 8
- 9
- 10 Pain as bad as you can imagine

Please rate your pain by selecting the one number that best describes your pain at its LEAST in the past 24 hours.

- 0 No pain
- 1
- 2
- 3
- 4
- 5
- 6
- 7
- 8
- 9
- 10 Pain as bad as you can imagine

Please rate your pain by selecting the one number that best describes your pain on AVERAGE.

- 0 No pain
- 1
- 2
- 3
- 4
- 5
- 6
- 7
- 8
- 9
- 10 Pain as bad as you can imagine

Please rate your pain by selecting the one number that tells how much pain you have RIGHT NOW.

- 0 No pain
- 1
- 2
- 3
- 4
- 5
- 6
- 7
- 8
- 9
- 10 Pain as bad as you can imagine

Item from Brief Pain Inventory © Copyright 1991 – Charles S. Cleeland, PhD Pain Research Group - All rights reserved.

| Page Break |  |
| --- | --- |

In the past 24 hours, how much RELIEF have pain treatment or medications provided? Please select the one percentage that best shows how much relief you have received.

- 0% No relief
- 10%
- 20%
- 30%
- 40%
- 50%
- 60%
- 70%
- 80%
- 90%
- 100% Complete relief

Item from Brief Pain Inventory © Copyright 1991 – Charles S. Cleeland, PhD Pain Research Group - All rights reserved.

| Page Break |  |
| --- | --- |

Select the one number that describes how, during the past 24 hours, PAIN HAS INTERFERED with your:

A. General activity

- 0 Does not interfere
- 1
- 2
- 3
- 4
- 5
- 6
- 7
- 8
- 9
- 10 Completely interferes

B. Mood

- 0 Does not interfere
- 1
- 2
- 3
- 4
- 5
- 6
- 7
- 8
- 9
- 10 Completely interferes

C. Walking ability

- 0 Does not interfere
- 1
- 2
- 3
- 4
- 5
- 6
- 7
- 8
- 9
- 10 Completely interferes

D. Normal work (includes both outside the home and housework)

- 0 Does not interfere
- 1
- 2
- 3
- 4
- 5
- 6
- 7
- 8
- 9
- 10 Completely interferes

E. Relations with other people

- 0 Does not interfere
- 1
- 2
- 3
- 4
- 5
- 6
- 7
- 8
- 9
- 10 Completely interferes

F. Sleep

- 0 Does not interfere
- 1
- 2
- 3
- 4
- 5
- 6
- 7
- 8
- 9
- 10 Completely interferes

G. Enjoyment with life

- 0 Does not interfere
- 1
- 2
- 3
- 4
- 5
- 6
- 7
- 8
- 9
- 10 Completely interferes

Item from Brief Pain Inventory © Copyright 1991 – Charles S. Cleeland, PhD Pain Research Group - All rights reserved.

| Page Break |  |
| --- | --- |

The Brief Illness Perception Questionnaire (Modified)
For the following questions, please select the number that best corresponds to your views:

How much does your pain affect your life?

- 0	no affect at all
- 1
- 2
- 3
- 4
- 5
- 6
- 7
- 8
- 9
- 10 severely affects my life

How long do you think your pain will continue?

- 0 a very short time
- 1
- 2
- 3
- 4
- 5
- 6
- 7
- 8
- 9
- 10 forever

How much control do you feel you have over your pain?

- 0 absolutely no control
- 1
- 2
- 3
- 4
- 5
- 6
- 7
- 8
- 9
- 10 extreme amount of control

How much do you think your treatment can help your pain?

- 0 not at all
- 1
- 2
- 3
- 4
- 5
- 6
- 7
- 8
- 9
- 10 extremely helpful

How much do you experience symptoms from your pain?

- 0 no symptoms at all
- 1
- 2
- 3
- 4
- 5
- 6
- 7
- 8
- 9
- 10 many severe symptoms

How concerned are you about your pain?

- 0 not at all concerned
- 1
- 2
- 3
- 4
- 5
- 6
- 7
- 8
- 9
- 10 extremely concerned

How well do you feel you understand your pain?

- 0 don't understand at all
- 1
- 2
- 3
- 4
- 5
- 6
- 7
- 8
- 9
- 10 understand very clearly

How much does your pain affect you emotionally? (e.g. does it make you angry, scared, upset or depressed?)

- 0 not at all affected emotionally
- 1
- 2
- 3
- 4
- 5
- 6
- 7
- 8
- 9
- 10 extremely affected emotionally

Reproduced with permission from Dr. Elizabeth Broadbent.

| Page Break |  |
| --- | --- |

Have you seen a health care professional about your pelvic pain?

- Yes
- No

What age did you first see a health care professional for your pelvic pain?

- Less than 18 years old
- 18
- 19
- 20
- 21
- 22
- 23
- 24
- 25
- 26
- 27
- 28
- 29
- 30
- 31
- 32
- 33
- 34
- 35
- 36
- 37
- 38
- 39
- 40
- 41
- 42
- 43
- 44
- 45
- 46
- 47
- 48
- 49
- 50
- 51
- 52
- 53
- 54
- 55
- 56
- 57
- 58
- 59
- 60
- 61
- 62
- 63
- 64
- 65
- 66
- 67
- 68
- 69
- 70
- 71
- 72
- 73
- 74
- 75
- 76
- 77
- 78
- 79
- 80
- 81
- 82
- 83
- 84
- 85
- 86
- 86
- 88
- 89
- 90
- 91
- 99
- 93
- 94
- 95
- 96
- 97
- 98
- 99
- 100 and Older

| Page Break |  |
| --- | --- |

During assessment of my pelvic pain, I felt the health care professional validated my pain and listened to my story.

- Strongly agree
- Somewhat agree
- Neither agree nor disagree
- Somewhat disagree
- Strongly disagree

I was asked what I thought the source of my symptoms or cause of my pain was.

- Strongly agree
- Somewhat agree
- Neither agree nor disagree
- Somewhat disagree
- Strongly disagree

I have received a diagnosis for my pelvic pain.

- Strongly agree
- Somewhat agree
- Neither agree nor disagree
- Somewhat disagree
- Strongly disagree

My pelvic pain has been correctly diagnosed.

- Strongly agree
- Somewhat agree
- Neither agree nor disagree
- Somewhat disagree
- Strongly disagree

Have you had a physical examination to complete the diagnosis? This may include abdominal, pelvic, vaginal or rectal examination/s.

- Yes
- No

I felt the physical examination was thorough enough to help with my diagnosis.

- Strongly agree
- Somewhat agree
- Neither agree nor disagree
- Somewhat disagree
- Strongly disagree

I felt the physical examination was helpful in my management or treatment.

- Strongly agree
- Somewhat agree
- Neither agree nor disagree
- Somewhat disagree
- Strongly disagree

Please select the investigations or procedures you have had done to get a diagnosis.

- Blood tests
- Urine test
- Vaginal swabs
- Biopsy (sample of tissue)
- Imaging, for example; CT, MRI, Ultrasound
- Scope or camera view for example; colonoscopy, cystoscopy, endoscopy
- Surgical procedure for example laparoscopy
- Other
- None of the above

During one of my appointments for pelvic pain the health care professional has explained that pelvic pain can have multiple causes and factors.

- Strongly agree
- Somewhat agree
- Neither agree nor disagree
- Somewhat disagree
- Strongly disagree

What do you believe the cause of your pain is? 
You may select more than one.

- Bladder
- Bowel
- Uterus
- Ovaries
- Pelvic floor muscles
- Ligaments or bone
- Stress
- Anxiety or depression
- Sleep deprivation or fatigue
- Nerves/Nervous system
- Infection
- Immune system
- Period/Menstrual pain
- Cancer
- Pathological condition: Endometriosis, Crohn's Disease, Interstitial cystitis etc
- Syndrome: Bladder Pain Syndrome, Irritable Bowel Syndrome etc
- Inflammation
- None of the above

I believe that there is something 'worse' happening to me that has not been diagnosed yet.

- Strongly agree
- Somewhat agree
- Neither agree nor disagree
- Somewhat disagree
- Strongly disagree

I believe a diagnosis or 'name' for my pelvic pain symptoms is important prior to participating in treatment.

- Strongly agree
- Somewhat agree
- Neither agree nor disagree
- Somewhat disagree
- Strongly disagree

| Page Break |  |
| --- | --- |

What do you believe can cause pelvic pain? 
You may select more than one.

- Bladder
- Bowel
- Uterus
- Ovaries
- Pelvic floor muscles
- Ligaments or bone
- Stress
- Anxiety or depression
- Sleep deprivation or fatigue
- Nerves/Nervous system
- Infection
- Immune system
- Period/Menstrual pain
- Cancer
- Pathological condition: Endometriosis, Crohn's Disease, Interstitial cystitis etc
- Syndrome: Bladder Pain Syndrome, Irritable Bowel Syndrome etc
- Inflammation
- All of the above

From the list below please select who you have seen for your pelvic pain.

- General Practitioner (GP)
- Gynaecologist
- Urologist
- Colorectal surgeon
- Gastroenterologist
- Pain specialist/Pain Physician
- Psychiatrist/Psychologist
- Physiotherapist
- Nutritionist/Dietitian
- Sexologist/Sexual Therapist
- Alternative Medicine (Chinese medicine, Chiropractor, Naturopath, Remedial Massage etc)
- ⊗None, I have not seen any of the above

From the same list, please select who has been helpful in the management of your pelvic pain.

- General Practitioner/GP
- Gynaecologist
- Urologist
- Colorectal surgeon
- Gastroenterologist
- Pain specialist/ Pain Physician
- Psychiatrist/Psychology
- Physiotherapist
- Nutritionist/Dietitian
- Sexologist/Sexual Therapist
- Alternative medicine (Chinese medicine, Chiropractor, Naturopath, Remedial Massage etc)
- None of the above have been helpful

Accessing treatment/care for my pelvic pain was difficult.

- Strongly agree
- Agree
- Neither agree nor disagree
- Somewhat disagree
- Strongly disagree

What do you believe have been the barriers in receiving care for your pelvic pain? 
You may select more than one

- Long wait lists
- Lack of information to make decisions about my care
- Time poor and unable to commit to management options
- Financial restraints and cost of management or treatment
- Lack of access to multiple health care practitioners
- Not understanding who I should see
- Not understanding my condition
- Management not helpful in the past so not willing to try again
- There have been no barriers

| Page Break |  |
| --- | --- |

Do you believe you need further investigations or procedures done to correctly diagnose your symptoms?

- Yes
- No
- Unsure

Please select those investigations or procedures you feel you require.

- Blood tests
- Urine tests
- Vaginal swabs
- Biopsy (sample of tissue)
- Imaging, for example; CT. MRI, Ultrasound
- Scope or camera view, for example; colonoscopy, cystoscopy, endoscopy
- Surgical procedure, for example a laparoscopy
- Other

| Page Break |  |
| --- | --- |

Pain medications help me manage my pain.

- Strongly agree
- Somewhat agree
- Neither agree nor disagree
- Somewhat disagree
- Strongly disagree

Hormonal treatment (oral contraceptive pill, mirena etc) help me manage my pain.

- Strongly agree
- Somewhat agree
- Neither agree nor disagree
- Somewhat disagree
- Strongly disagree

From the list below, please select any management strategies that have helped manage your pelvic pain.

- Avoid activity, exercise or tasks
- Meditation/Mindfulness
- Breathing and relaxation exercises
- Psychological or behavioural therapy
- Heat pack or cool pack
- Low impact paced exercise (walking, cycling, swimming, light weights)
- High intensity exercise (running, HITT class, aerobics, cardio gym class)
- Strengthening exercises (Pilates, weights, gym)
- Stretching or Yoga
- Playing Sport
- Dancing
- Carrying out good bladder and bowel habits
- Dietary assistance (low FODMAP, low sugar, low processed food, gluten free, vegan)
- Socialising or connecting with friends and family
- Pelvic Physiotherapy (prescribed exercises, soft tissue massage of pelvic muscles, dilator therapy, electrical stimulation, dry needling)
- Massage therapy
- Electrical stimulation (TENS machine) or Implanted device
- Acupuncture or Chinese Medicine
- Naturopathy or herbal medicine
- Tai Chi/Qi Gong
- Alcohol
- Cannabis or hemp oil
- Surgery or procedures
- Hobbies: music, art, drawing etc

Please rank the following management strategies you believe are most helpful for those living with pelvic pain.

|  | Not helpful | Unsure | Helpful |
| --- | --- | --- | --- |
| Avoid activity/exercise/tasks |  |  |  |
| Meditation/Mindfulness |  |  |  |
| Breathing and relaxation exercises |  |  |  |
| Psychological or behavioural therapy |  |  |  |
| Heat or cool pack |  |  |  |
| Low impact paced exercise (walking, cycling, swimming, light weights) |  |  |  |
| High intensity exercise (running, HITT class, aerobics, cardio gym class) |  |  |  |
| Strengthening exercises (pilates, weights, gym) |  |  |  |
| Stretching or Yoga |  |  |  |
| Playing sport |  |  |  |
| Dancing |  |  |  |
| Carrying out good bladder and bowel habits |  |  |  |
| Dietary assistance (low FODMAP, low sugar, low processed food, gluten free, vegan) |  |  |  |
| Socialising or connecting with friends and family |  |  |  |
| Pelvic Physiotherapy (prescribed exercises, soft tissue massage of pelvic muscles, dilator therapy, electrical stimulation, dry needling) |  |  |  |
| Massage therapy |  |  |  |
| Electrical stimulation (TENS machine) or Implanted device |  |  |  |
| Acupuncture or Chinese Medicine |  |  |  |
| Naturopathy or herbal medicine |  |  |  |
| Tai Chi/Qi Gong |  |  |  |
| Alcohol |  |  |  |
| Cannabis or hemp oil |  |  |  |
| Surgery or procedure |  |  |  |
| Hobbies: music, art, drawing etc |  |  |  |

I feel socially supported and/or understood by family, partner or friends when I am in pain.

- Strongly agree
- Somewhat agree
- Neither agree nor disagree
- Somewhat disagree
- Strongly disagree

| Page Break |  |
| --- | --- |

Disclosure: We realise some women with pelvic pain have experienced sexual abuse in the past. Please know help is available if these questions raise concerns for you. 

If you have experienced sexual abuse and you would like to speak to someone for confidential and anonymous counselling and support, you can call the following numbers anywhere in Australia 24 hours a day, 7 days a week. 1800RESPECT (Phone: 1800 737 732)  Lifeline 13 11 14 Or you can get further information for services from: www.respect.gov.au www.lifeline.org.au www.healthdirect.gov.au/sexual-assault-and-abuse-helplines www.rape-dvservices.org.au www.anrows.org.au If you, or another person are in immediate danger, call triple zero (000).  
The following questions relate to sexual abuse. If you do not want to answer these questions, we thank you for your time and you can complete the survey here. Otherwise, please answer the following questions only if you are comfortable in doing so. 
Do you wish to continue?

- Yes
- No

| Page Break |  |
| --- | --- |

Have you experienced sexual abuse in the past?

- Yes
- No
- Prefer not to answer

Has any health care professional ever asked you the previous question before?

- Yes
- No
- Prefer not to answer

Do you feel your sexual abuse experience influences your current pelvic pain levels?

- Yes
- Maybe
- No
- Unsure

If you have experienced sexual abuse and you would like to speak to someone for confidential and anonymous counselling and support, you can call the following numbers anywhere in Australia 24 hours a day, 7 days a week.  1800RESPECT (Phone: 1800 737 732)   Lifeline 13 11 14 Or you can get further information for services from: www.respect.gov.au www.lifeline.org.au www.healthdirect.gov.au/sexual-assault-and-abuse-helplines www.rape-dvservices.org.au www.anrows.org.au If you, or another person are in immediate danger, call triple zero (000).

End of Block: Block 1
